# Tenth Annual Meeting of the American Society of Dental Surgeons

**Published:** 1850-07

**Authors:** A. Westcott


From the Am. Jour. of Dental Science.

### TENTH ANNUAL MEETING OF THE AMERICAN SOCIETY OF DENTAL SURGEONS;

HELD IN THE CITY OF BALTIMORE, THE 25th, 26th, and 27th DAYS OF MARCH, 1850.

THE Society convened at the Lecture Hall of the Baltimore Dental College, on the 25th of March, 1850, at 12 M.

The first business transacted was the appointment of Committees, as follows:

1st. Committee to arrange the order of business.—DRS. A. WESTCOTT, C. O. CONE, R. ARTHUR.

2d. Committee on grievances, to examine and report upon papers pertaining to the moral standing of one of the members. On this Committee, the President appointed—DRS. CLEVELAND, TOWNSEND, FOSTER. To which were added by nomination, E. PARMLY, C. A. HARRIS.

The Recording Secretary here asked to be excused from keeping any minutes (for publication) relating to the business of the first named Committee, and moved that Dr. R. Arthur be substituted to keep the minutes pertaining to this subject. Dr. Arthur was so appointed. Adjourned till nine o'clock tomorrow.

BALTIMORE DENTAL COLLEGE, March 26th, 1850.
9 o'clock A. M.

The Society convened agreeably to adjournment.

The Committee on arrangements reported the following order:

1st. Reading by Dr. Harris, a Memoir of the late Dr. Nasmyth.

2d. Report of Committee on Grievances.

3d. " " Dental Education.

4th. " " Practical Dentistry.

5th. " " Tabular Sheet or Dental Record.

6th. Report of Committee on Treasurer.
7th. " " Condition of the Journal.
8th. " " Discussions on Practical Subjects.

In pursuance of the above order, at ten o'clock, Dr. Harris read the Memoir of Dr. Nasmyth.

On motion of C. O. Cone, the thanks of the Society were presented to Dr. H., for said Memoir, and a copy requested for publication.

The reading of this paper elicited appropriate remarks from several members present, particularly from Dr. E. Parmly, after which, on motion of Dr. Foster, the Society tendered their thanks to Mrs. Nasmyth for presenting to the profession the result of the researches of her late husband.

The committee on Grievances, in the case referred to them, reported progress.

Dr. R. Arthur, from the committee on Dental Education, (appointed August, 1848,) reported that the duties of the committee had not been fully understood, and hence, nothing had been done. He therefore moved that the committee be dismissed, and that the subject be divided and appropriate committees be appointed to take charge of the respective branches.

The old committee was dismissed and Dr. Arthur was requested to name the divisions and define them. Dr. A. then read a paper on this subject, which gave rise to some discussion, in which Dr. Arthur, Hullihen, and several others participated. It was finally agreed that the division made should be into *Dental Literature* and confined to *American Literature*, and Dental Education.

Persons appointed to prepare a paper, on the former, Dr. R. Arthur, on the latter, A. Westcott.

The committee on Practical Dentistry was called, but none of its members being present, the subject was, on motion, laid over, and the former committee, consisting of Dr. J. B. Rich, E. Maynard and Alexander Nelson, was continued.

Dr. E. Townsend here gave notice, that he wished at a proper time to present a subject to the Society, on which a committee might be called for. He wished also, to present some other promiscuous business. It was therefore moved to suspend the regular order, pro tem.

Dr. T. then presented an application for membership from Dr. J. D. White, of Philadelphia.

He then, after some introductory remarks, proceeded to read a paper which he had prepared for the Society. This paper was upon the subject of the "*Amalgam Pledge*," and after remarks from most of the members present, was, on motion of A. Westcott, seconded by Dr. J. H. Foster, referred to a committee, to report upon the subject matter at the next meeting of the Society.

The committee was appointed by the chair, and was as follows:—A. Westcott, E. Townsend, J. H. Foster.

The Treasurer's books were received, (the Treasurer, E. J. Dunning, not being present,) and a committee was appointed to examine and report upon the same, as follows:—Drs. Harris, Cone and Arthur.

A committee was also appointed to report at the next meeting upon the condition, prospects and future management of the Journal, as follows:—Drs. Harris, Westcott, Townsend, to which was added Drs. E. Parmly and Cone.

A resolution was offered by Dr. Foster, directing the Recording Secretary to inquire of the Corresponding Secretary, why he had so long retained the report of the last meeting, (made by Mr. Burr, reported,) thereby preventing the publication of the same, carried. Adjourned till to-morrow.

Baltimore Dental College, Feb, 27th. 10 o'clock, A. M.

Dr. C. O. Cone made a verbal report on the "Tabular Sheet"—said it was not yet fully completed. On motion, he was requested to complete the same and report at next meeting.

Dr. Cone, from the committee on the Journal, made a report which was ordered to be placed on file. Dr. Harris made a proposition to take a certain amount of the back numbers of our Journals towards the money he had advanced. His proposition was accepted and ordered to be placed on file.

On motion, Dr. Harris was authorized to collect dues for Journal and for membership.

Dr. L. S. Parmly was authorized to act as general agent for the Society, both in this country and in Europe.

Dr. W. H. Dwindle withdrew as an editor of the Journal, and Dr. E. Maynard, of Washington City, was appointed in his place.

On motion of A. Westcott, a committee was appointed to revise the Constitution, particularly that portion of it pertaining to the admission of members. The committee is as follows:—Drs. Westcott, Hullihen and Dunning.

On motion, it was resolved, that the officers of this Society, (except in cases of vacancy,) hold over till the next annual meeting.

Dr. S. P. Hullihen moved that a cammittee be appointed to prepare an address to the *public*, carried. Dr. S. P. Hullihen was appointed as such committee.

*Discussions in Practical Dentistry.*—The subject of filling over exposed nerves without destroying them, was discussed at considerable length, Dr. Hullihen exhibited the kind of cap which he employed, and showed the manner of making and using it.

Dr. H. also exhibited a new method of putting teeth on to plate, also several ingenious fixtures used in regulating teeth.

Dr. Cleveland exhibited a new method of constructing air chambers in plates, showing great ingenuity and skill, both in design and execution. The general subject of cavity or air-chamber plates was very fully discussed.

Dr. E. Townsend exhibited fixtures for regulating teeth, and explained their mode of application.

*Adjourned to meet on the second Tuesday of August*, 1850, *at Saratoga Springs.*

A. Westcott, Recording Secretary.

